# An ecohydrological journey of 4500 years reveals a stable but threatened precipitation–groundwater recharge relation around Jerusalem

**DOI:** 10.1126/sciadv.abe6303

**Published:** 2021-09-10

**Authors:** Simone Fatichi, Nadav Peleg, Theodoros Mastrotheodoros, Christoforos Pappas, Gabriele Manoli

**Affiliations:** 1 Department of Civil and Environmental Engineering, National University of Singapore, 1 Engineering Drive 2, 117576 Singapore, Singapore.; 2 Institute of Environmental Engineering, ETH Zurich, Stefano-Franscini-Platz 5, 8093 Zurich, Switzerland.; 3 Institute of Earth Surface Dynamics, University of Lausanne, Géopolis, 1015 Lausanne, Switzerland.; 4 Centre d’étude de la forêt, Université du Québec à Montréal, C.P. 8888, Succursale Centre-ville, Montréal, QC H3C 3P8, Canada.; 5 Département Science et Technologie, Téluq, Université du Québec, 5800 rue Saint-Denis, Bureau 1105, Montréal, QC H2S 3L5, Canada.; 6 Department of Civil, Environmental and Geomatic Engineering, University College London, Gower Street, WC1E 6BT London, UK.

## Abstract

Groundwater is a key water resource in semiarid and seasonally dry regions around the world, which is replenished by intermittent precipitation events and mediated by vegetation, soil, and regolith properties. Here, a climate reconstruction of 4500 years for the Jerusalem region was used to determine the relation between climate, vegetation, and groundwater recharge. Despite changes in air temperature and vegetation characteristics, simulated recharge remained linearly related to precipitation over the entire analyzed period, with drier decades having lower rates of recharge for a given annual precipitation due to soil memory effects. We show that in recent decades, the lack of changes in the precipitation–groundwater recharge relation results from the compensating responses of vegetation to increasing CO_2_, i.e., increased leaf area and reduced stomatal conductance. This multicentury relation is expected to be modified by climate change, with changes up to −20% in recharge for unchanged precipitation, potentially jeopardizing water resource availability.

## Teaser

Climate change can alter Jerusalem’s precipitation–groundwater recharge relation, which has been stable for 4500 years.

## INTRODUCTION

Depletion of groundwater storage has been reported in several regions worldwide (*1*, *2*), and while groundwater pumping is one of the main reasons (*3*), changes in climatic variables might also have a considerable role on groundwater storage and recharge (*4*, *5*). Groundwater recharge is the residual of precipitation minus the sum of evapotranspiration (ET) and runoff and hence is strongly affected by climate variability, vegetation, soil, and regolith properties (*6*). In semiarid regions, even relatively minor changes in ET can strongly influence groundwater recharge, as recharge is a small flux in comparison to precipitation and ET, and runoff is also a tiny fraction of precipitation (*7*).

Although small in magnitude, groundwater recharge in semiarid regions is key for water resource management as it replenishes deep aquifers that, in turn, through perennial springs and/or through direct pumping, provide the only continuous source of water (*8*, *9*). In this regard, the impact of water availability on the thriving and decay of early civilizations living in semiarid or seasonally dry regions has been often reported (*10*, *11*). In particular in the Levant, links between precipitation and societal changes have been documented (*12*–*15*). In these regions, the first few meters of soil (vadose zone) are an important control of groundwater recharge amount, as geological and deep subsurface properties mainly control the timing of aquifer refill, the flow patterns, and the seasonality of spring discharge (*7*). Yet, studies on groundwater recharge generally focused on the role of geologic formation or soil properties on water flow (*16*–*18*), with less emphasis on vegetation and its impact on ET. Even ecohydrological studies examining the role of vegetation on groundwater recharge either focus on quantifying the amount of plant water uptake directly from groundwater (*19*, *20*) or are limited to relatively short time scales (few years or decades). A broader perspective on the role of vegetation in mediating groundwater recharge fluxes necessitates longer time scales, which are also extremely useful to frame the effects of relatively recent anthropogenic climate change in a multicentury perspective.

Here, we analyze the role of vegetation and soil moisture dynamics on groundwater recharge with a mechanistic ecohydrological model (*21*, *22*), validated by reproducing groundwater recharge observations in the Jerusalem region (see Materials and Methods), rather than focusing on aquifer properties and saturated flow in deep geological formations. We use a recently reconstructed 4500-year time series of annual precipitation for the Jerusalem area (*15*), paleoclimate reconstructions using proxies (*23*, *24*) and models (*25*, *26*) for other climatic variables, and an hourly weather generator (*27*, *28*). This allows extending the analysis of groundwater recharge to temporal scales that are typical of paleoclimate studies but unprecedented in mechanistic ecohydrological studies (see Materials and Methods). With these model simulations, we address the following questions: (i) Has the relation between precipitation and groundwater recharge been affected by other variables during the past 4500 years?; and (ii) how is the relation between precipitation and groundwater recharge going to change in the future with increasing temperature and elevated CO_2_? Answering these questions sheds light on the long-term linkage between vegetation dynamics and groundwater recharge and can provide guidance for reliable water resources management under climate change in the Jerusalem area and, more generally, in other semiarid regions characterized by seasonal precipitation.

## RESULTS

### 4500 years of ecohydrological data

**Fig. 1.**
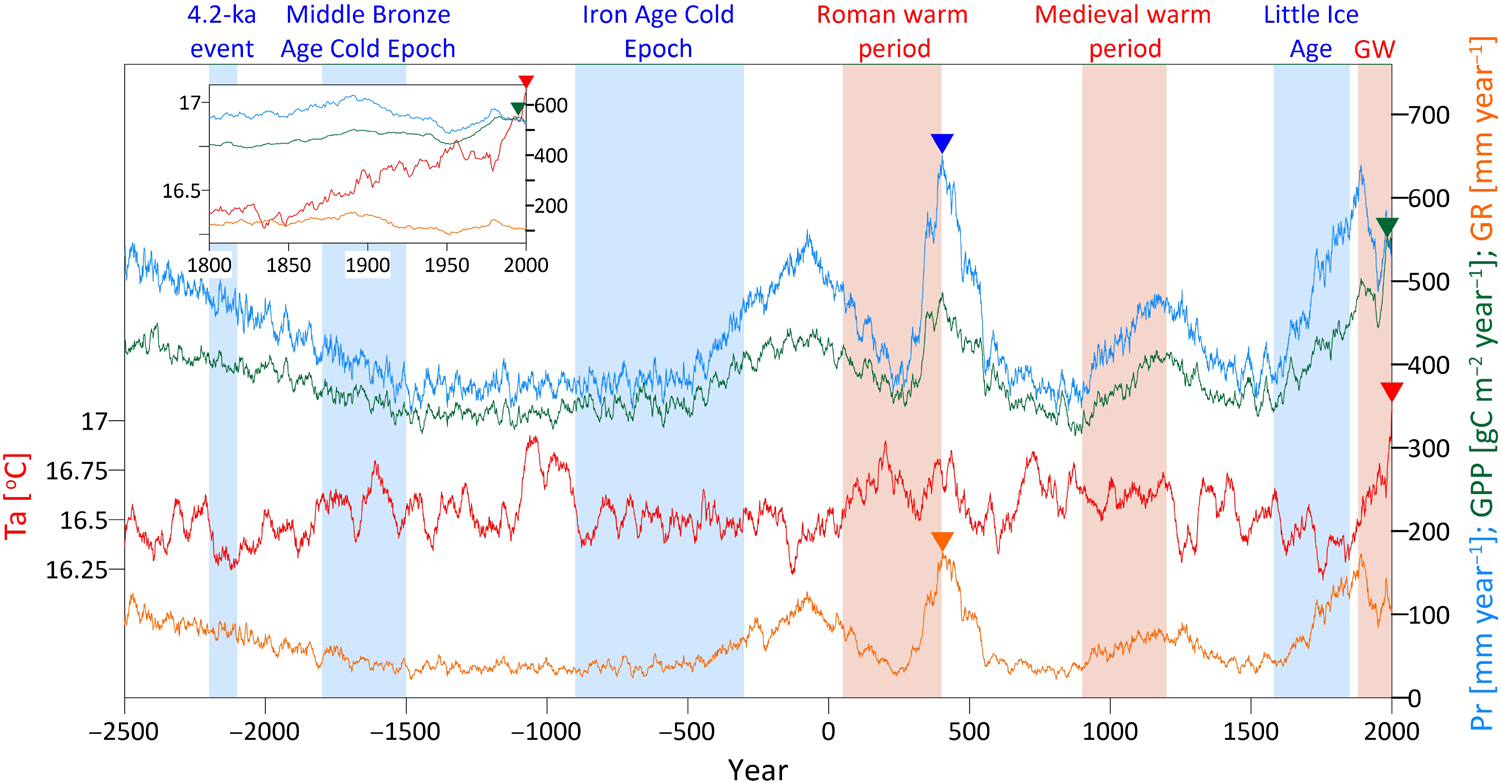
Time series of meteorological forcing and simulated ecohydrological responses. The 30-year moving average of reconstructed meteorological forcing [near-surface air temperature (Ta), red line; precipitation (Pr), blue line] and simulated vegetation GPP (green line) and groundwater recharge (GR, orange line) between 2500 BCE and 2000 CE. Triangles show the maximum values of each variable in the time series. Major known cold and warm climatic epochs are framed in blue and red areas (respectively); GW, the current Global Warming. The past 200 years are zoomed in the inset.

The combination of paleoclimate reconstructions, a weather generator, and a mechanistic ecohydrological model allowed for the simulation of more than 4500 years of hourly water and carbon dynamics over a grass/shrub biome typical of natural vegetation surrounding the Jerusalem area. Reconstructed meteorological forcing (precipitation and air temperature) and simulated vegetation gross primary production (GPP) and groundwater recharge (GR) reflect the signature of major climatic events that occurred in the past 4500 years, such as the Iron Age Cold Epoch, the Roman and Medieval warm periods, the Little Ice Age, and the recent global warming, which is very evident in the temperature time series, especially in the past 50 years (Fig. 1). In this region, colder periods of the past are typically associated with drier conditions and vice versa as often observed in geological records (*29*) and also in global model projections (*30*). The highest precipitation in the record is during the Roman warm period when the 30-year average precipitation exceeded 650 mm year^−1^. Recharge patterns follow closely precipitation patterns with recharge decreasing from ~100 mm year^−1^ to roughly 30 to 35 mm year^−1^ in the first 1000 years, increasing back to 120 mm year^−1^ around year 75 BCE and with oscillations leading to the highest 30-year average recharge peaks (~170 mm year^−1^) in the years 402–415 CE and low values ~30 to 50 mm year^−1^ in the 650–900 CE and 1400–1620 CE. The past 200 years are characterized by an initial positive (up to 1900 CE) and then a negative trend in recharge with an average value of 128 mm year^−1^. A negative trend (−1.7 mm year^−2^) is simulated in the past 20 years. Vegetation is responsive to climatic fluctuations and especially to increase in precipitation with a peak in GPP during the Roman warm period at 485 gC m^−2^ year^−1^. GPP is increasing since 1800 CE first in response to increased precipitation, but then in the past 20 years, when precipitation trends have reversed, GPP has still increased or remained stable in response to CO_2_. This is because of CO_2_ stimulation of photosynthesis and increasing trends in water use efficiency (WUE) (*31*, *32*). The 30-year average GPP reached a peak of 554 gC m^−2^ year^−1^ in 1983 and 1995 CE—unprecedented levels for the past 4500 years. Changes in GPP are associated with simulated changes in vegetation cover in terms of leaf area index (LAI), which on a 30-year average ranges between 0.6 and 0.9 in the simulated period (fig. S1), with a clear positive trend since the 1950s.

**Fig. 2.**
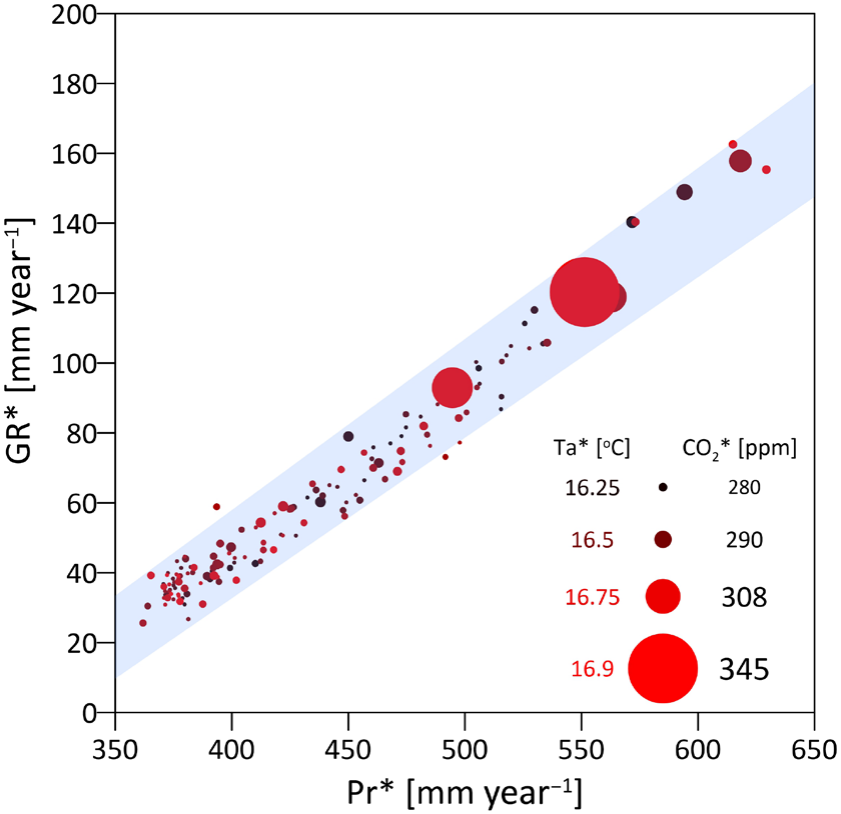
Precipitation–groundwater recharge relation across 4500 years. Mean 30-year groundwater recharge (GR*) as a function of the mean 30-year precipitation (Pr*) for the simulated period between 2500 BCE and 2000 CE [GR* = 0.47Pr*−144.5; *R*^2^ (coefficient of determination) = 0.96 and *P* < 0.0001]. Mean 30-year CO_2_ levels are represented by symbol size, while symbol colors represent the mean 30-year air temperature. The shaded area represents the 5 to 95% confidence interval of the Pr*-GR* linear regression.

The changes in air temperature and CO_2_ (and consequently vegetation) occurred mostly in the past 50 years but do not seem to considerably modify the relation between precipitation and groundwater recharge (Fig. 1). Even other components of the hydrological budget, once normalized with precipitation, do not show any consistent trend from 1850 to 2000 CE (fig. S2). This is supported by a relatively stable relation between mean 30-year precipitation (Pr*) and mean 30-year groundwater recharge (GR*) (Fig. 2). The stability of this relation for 4500 years, despite the fluctuations in other variables (fig. S3), underlines the dominant role of precipitation in controlling groundwater recharge. The effects of temperature and CO_2_ generate small deviations from this line, more distinct during the past 60 years (two 30-year blocks); however, only two 30-year intervals (191–220 BCE and 921–950 CE) are not within the 5 to 95% confidence interval of the linear Pr*-GR* relation (Fig. 2).

**Fig. 3.**
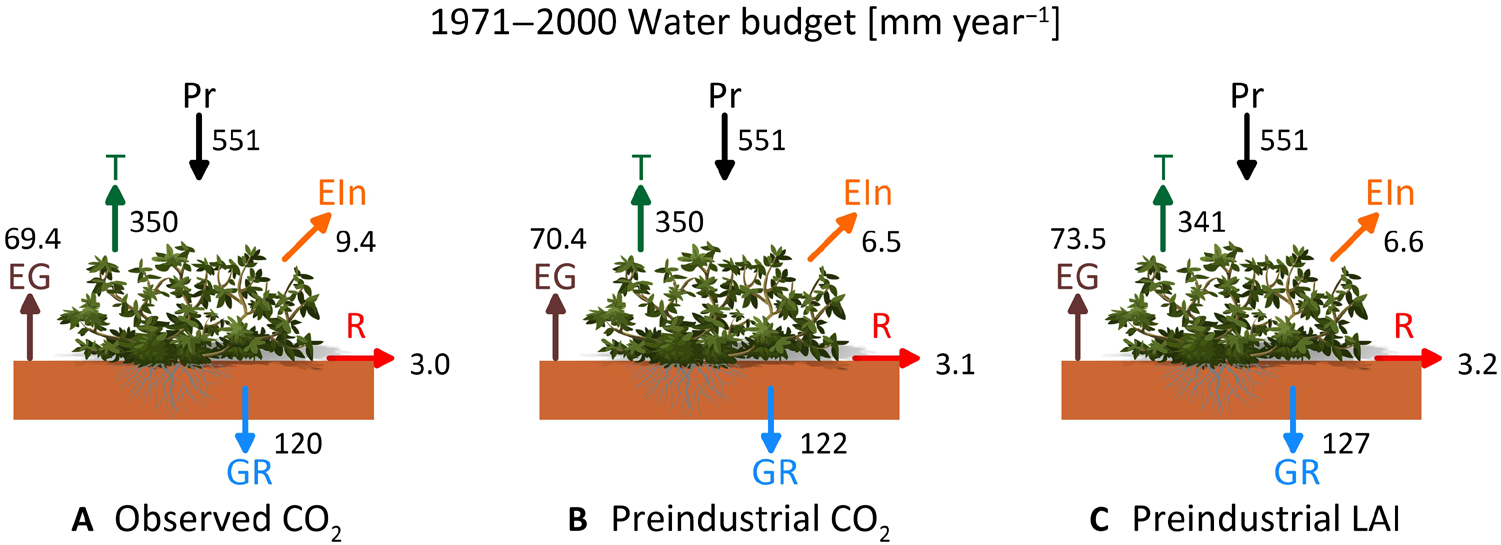
Hydrological budget components for the 1971–2000 period. The mean water budget components (millimeter per year) simulated with Tethys-Chloris forced with (**A**) AWE-GEN simulated meteorological variables for the period 1971–2000 CE (GPP, 545 gC m^−2^ year^−1^ and LAI, 0.97 m^2^ m^−2^); (**B**) same forcing but with CO_2_ fixed to preindustrial levels of 280 ppm (GPP, 455 gC m^−2^ year^−1^ and LAI, 0.83 m^2^ m^−2^); and (**C**) same forcing but with biomass pools (e.g., leaves, roots) kept to the preindustrial level (GPP, 510 gC m^−2^ year^−1^ and LAI, 0.83 m^2^ m^−2^). Pr, precipitation; T, transpiration; EG, ground evaporation, EIn, evaporation from interception; R, runoff; and GR, groundwater recharge.

The reason for the stability in the Pr*-GR* relation might be associated with the much larger fluctuations in precipitation (the variable with the highest interannual variability; Fig. 1) than for other meteorological variables (fig. S3). However, this does not provide a sufficient explanation for the past few decades when air temperature, CO_2_, and vegetation (GPP and LAI) changed considerably in comparison to the previous 4500 years. Therefore, the water budget of the last 30 simulated years (1971–2000 CE) was scrutinized further by computing the hydrological budget components and comparing with two hypothetical experiments where CO_2_ or biomass pools (e.g., leaves, fine roots) are kept to the preindustrial level (Fig. 3). Two compensating effects, an increase in LAI and a decrease in stomatal conductance, led to minor changes in transpiration despite a considerable increase in GPP and LAI and maintained stable groundwater recharge over this period. Increased LAI in response to increasing CO_2_ should lead to higher transpiration rates (per unit of ground) and lower recharge, but this is compensated by the physiological effect of stomatal closure with elevated CO_2_, which leads to water savings (i.e., less transpiration per unit of leaf area). When CO_2_ is kept to the preindustrial level (Fig. 3B), simulated groundwater recharge is similar (+1.4%) as vegetation does not grow as much as in the reference scenario (testified by lower evaporation from interception, −31%, and higher ground evaporation, +1.4%) but it does not close stomata either. However, if we allow only CO_2_ physiological effects and keep biomass pools, including LAI, to preindustrial levels (Fig. 3C), then the simulated transpiration and evaporation from interception are reduced (−2.6 and −29%, respectively) because LAI does not increase, which cascades in a considerable +5.5% change in groundwater recharge. These potential water savings do not actually occur because recent climate change stimulates production of leaves and a larger LAI, which implies that vegetation uses more water than would be expected from CO_2_-induced stomatal closure alone (*31*–*33*). The net result is that transpiration and groundwater recharge remain stable despite the non-negligible vegetation dynamics that occurred in the past 30 to 50 years.

**Fig. 4.**
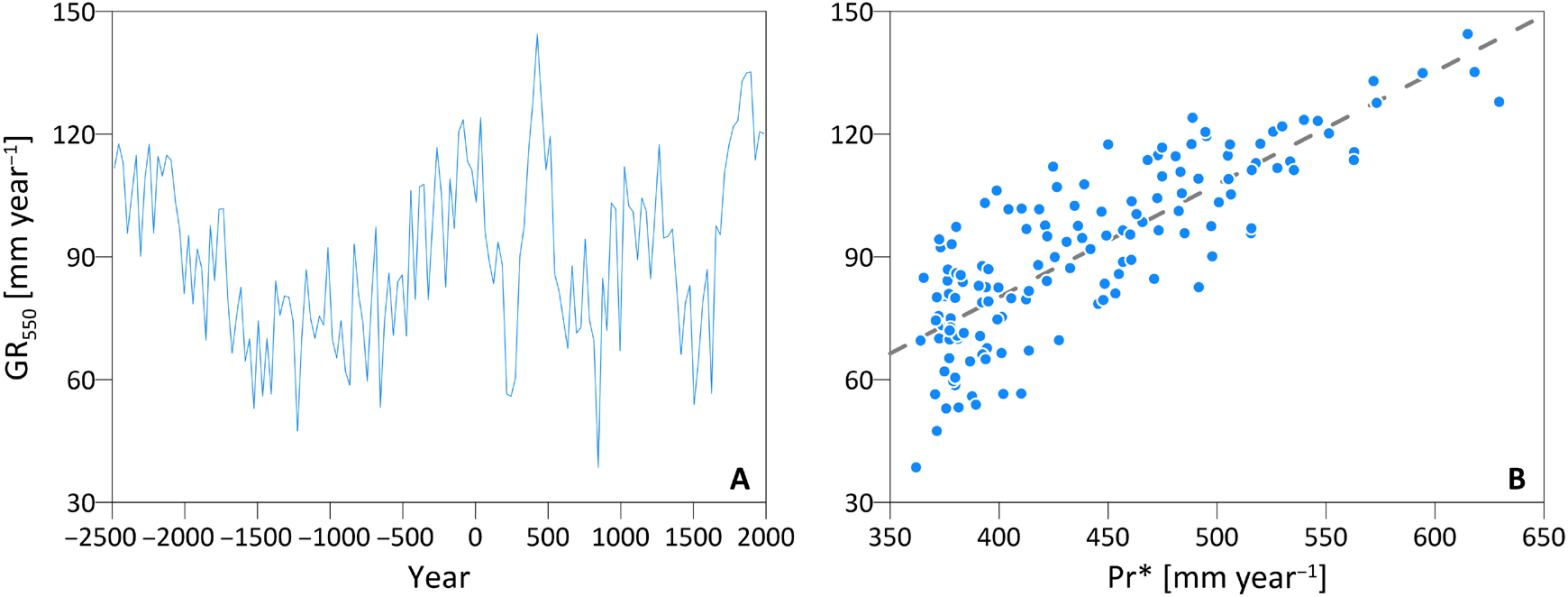
Variability in groundwater recharge for a reference annual precipitation. (**A**) Thirty-year mean annual groundwater recharge computed for a reference annual precipitation of 550 mm year^−1^ (GR_550_) between 2500 BCE and 2000 CE and (**B**) the linear relation (dashed line, *R*^2^ = 0.68 and *P* < 0.0001) between GR_550_ and the mean precipitation Pr* of each 30-year interval.

Stability of the Pr*-GR* relation over a 30-year time scale does not mean that at the annual scale recharge might not be affected by other climatic conditions or legacy effects. Specifically, we found a pronounced role of the mean precipitation of a 30-year period on the annual recharge for a given annual precipitation amount (Fig. 4). For each of the simulated 30-year periods between 2500 BCE and 2000 CE, we fit the annual Pr-GR relation and extract the GR value at the reference annual precipitation of today (550 mm year^−1^). This computed value, which corresponds to the same nominal amount of annual precipitation, varies through time quite considerably (Fig. 4A) and largely depends on the mean precipitation of the 30-year periods [*R*^2^ (coefficient of determination) = 0.68; Fig. 4B]. This supports the hypothesis that memory effects related to soil moisture, mostly driven by decadal precipitation, control the recharge on a multiannual scale, and that during wetter decades it is easier to recharge the aquifer for the same amount of annual precipitation.

### Sensitivity to CO_2_ and temperature increase

**Fig. 5.**
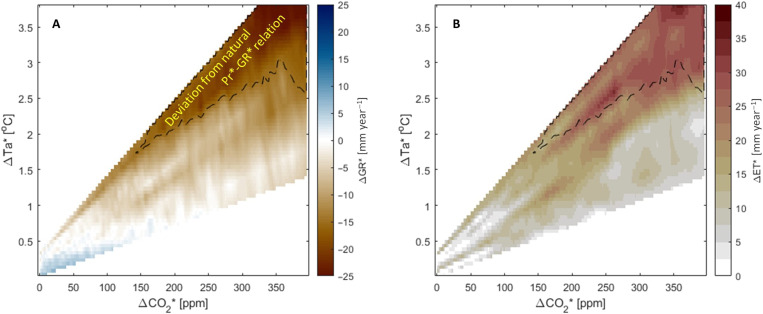
Sensitivity to CO_2_ and air temperature increase. (**A**) Changes from the historical Pr*-GR* relation (shown in Fig. 3) of groundwater recharge ΔGR* [millimeter per year], for several combinations of increasing Ta and CO_2_ (fig. S8). (**B**) Changes in ET, ΔET* [millimeter per year] from present-day conditions (ΔTa* = 0 and ΔCO_2_* = 0) for the same combinations of increasing Ta* and CO_2_* as in (A). The dashed line marks in both subplots the lower boundary of the area where estimates of GR* exceed the 5th to 95th confidence interval of the natural Pr*-GR* relation.

The changes in vegetation and temperature that occurred in the past 30 years raise concerns that the remarkable stability in the historical Pr*-GR* relation (Fig. 2) might be broken in the future with increasing air temperature (Ta) and CO_2_. To examine this, we analyzed the simulated deviations from the expected Pr*-GR* relation for several scenarios of temperature and CO_2_ increases (Fig. 5A). For scenarios with a small increase in temperature (by up to +0.4°C), we simulate recharge increases by up to +7.3 mm year^−1^ (+6.4%) from the historical Pr*-GR* relation. This is mostly the result of CO_2_-induced stomatal closure (fig. S4, A and B). However, for most combinations of increased Ta-CO_2_, a negative deviation from the historical recharge is detected (Fig. 5A), which might reach a change of −25 mm year^−1^ (corresponding to a −22% change from the expected Pr*-GR* relation). The stability of the “historical recharge” might break (i.e., GR* exceeds the 5th to 95th confidence interval of the historical relation) as soon as CO_2_ levels and Ta increase by 145 parts per million (ppm) and 1.8°C, respectively, compared with the means of the 1971–2000 CE period (Fig. 5A). Note that this is obtained for an unchanged Pr* of 550 mm year^−1^. Changes in groundwater recharge are mostly induced by changes in ET (Fig. 5B) and are more pronounced when the increase in Ta is above 2° to 2.5°C. Higher Ta increases transpiration and ground evaporation because of larger vapor pressure deficits (VPDs), even when changes in LAI are constrained by augmented water stress (fig. S4, A, C, and D). LAI increases considerably for a relatively small increase in Ta and a high increase in CO_2_. However, for fixed CO_2_, LAI decreases with increasing Ta (fig. S5B). This occurs because a larger winter transpiration (fig. S5A) caused by higher VPDs (fig. S5C) triggers an earlier onset of vegetation water stress at the end of the wet season (fig. S5D). A similar pattern toward increased water stress has been also documented in places where the growing season starts earlier (*34*). The net result is that climate change (higher Ta and CO_2_) can push the Pr*-GR* relation out of the historical envelope, decreasing considerably groundwater recharge even for unchanged precipitation.

## DISCUSSION

### The present

The importance of groundwater recharge in semiarid regions for water resources management is undisputable (*7*). In these regions, groundwater recharge emerges from the delicate balance between precipitation and ET, which are typically much larger than the groundwater recharge itself. Global warming has increased and will further increase the atmospheric demand for water over land (*35*), and rising CO_2_ has been postulated to contribute considerably to carbon uptake and “greening” of semiarid ecosystems (*36*). Both factors might increase ET and reduce groundwater recharge. However, we found little change in recharge over the past 30 years despite considerable changes in temperature and vegetation productivity. We show that this is the result of two contrasting and largely compensating roles that atmospheric CO_2_ exerts on vegetation. Increasing atmospheric CO_2_ has stimulated vegetation productivity and LAI not only in the model simulations but also in reality as observed in many semiarid regions (*31*). However, it has also increased WUE, decreasing leaf-level transpiration for a unit of assimilated carbon (*37*), as documented by modeling (*32*, *38*) and observational studies (*39*). In the Jerusalem case study, these two “biophysical” and “physiological” effects have largely canceled each other, leaving transpiration relatively unchanged in recent years (fig. S2). Furthermore, the recent increase in air temperature was not large enough to substantially modify VPD and consequently alter ground evaporation and transpiration. As precipitation has fluctuated but without a clear trend, this has largely preserved a stable recharge (~120 mm year^−1^; Fig. 1) over the past six decades in the Jerusalem region despite considerable climate change effects.

### The past

The 4500-year time span studied here is unique for detailed ecohydrological analyses [but see (*40*) for a more conceptual approach], which often focus on a few decades only. Under the assumption of natural land cover without species modification, reconstructing 4500 years of climate shows considerable changes in 30-year average recharge ranging from less than 40 mm year^−1^ during the Iron Age Cold Epoch and Little Ice Age, up to 160 mm year^−1^ during the Roman warm period and the late 19th century (Fig. 1). The results remark the overwhelming role of precipitation fluctuations for recharge found in other studies (*9*) with the other climate variables and vegetation dynamics creating only small deviations from an unexpectedly stable Pr*-GR* relation (only two statistically significant deviations in a simulated record of 150 × 30-year periods; Fig. 2). Such stability is the result of (i) the much larger variability of precipitation in comparison to other climatic variables across centuries (Fig. 1 and fig. S3); and (ii) the strong seasonality of precipitation, typical of Mediterranean climate, with most of the groundwater recharge occurring in winter when temperatures are lower and vegetation is less active than in spring, i.e., the system is energy-limited (fig. S6). Although vegetation responds by increasing GPP and LAI in wetter epochs (Fig. 1), it is not able to fully exploit the additional amount of water available and convert it into ET as precipitation mostly occurs in the winter and favors recharge rather than transpiration (fig. S6). Weather simulations over 4500 years produce many different combinations of precipitation events and interannual variability, but they do not generate systematic shifts in the precipitation seasonality, which are not documented and thus not implemented in the weather generator. If occurred, then changes in seasonality could potentially modify this pattern. Precipitation is not only dominant in determining the average recharge over 30 years but there is also a strong correlation between annual precipitation and annual recharge. Simulations show that soil moisture memory effects, exemplified by the mean long-term precipitation (Fig. 4B), modify the expected value of recharge for a given amount of annual precipitation, i.e., the recharge for a nominal annual precipitation is higher during wetter periods. Although we simply consider a 2-m soil profile, soil moisture effects in deeper layers (>1 m) of the soil profile persist for years and overwhelm the contribution of larger vegetation biomass in wet periods, which should rather generate a negative feedback on recharge.

### The future

The sensitivity analysis to changes in air temperature and atmospheric CO_2_ for fixed average precipitation (Fig. 5) shows that while deviations from the historical Pr*-GR* relation over natural vegetation have not occurred in the past, they can occur in the future. Deviations of up to −25 mm year^−1^ from the expected recharge of ~120 mm year^−1^ for a 550 mm year^−1^ precipitation are simulated by the model for warming exceeding 2°C. While recharge of 80 to 100 mm year^−1^ is not unprecedented in the past 4500 years, as several centuries had lower recharge rates (down to 30 mm year^−1^), they will be completely unprecedented for a 550 mm year^−1^ precipitation. This implies that while climate change has not pushed the response of the natural system out of the historical envelope yet, it can do so in the future. If these changes are combined with negative fluctuations in precipitation [plausible for the Jerusalem region (*41*) although much harder to predict than CO_2_ or Ta changes], then groundwater recharge might be reduced to values never experienced in the past 4500 years with clear implications for water resource management. Mitigating these impacts will demand preventing CO_2_ to rise to critical values and/or diversifying water supply sources. If the increase in CO_2_ is accompanied by only moderate warming below 1.5°C (and assuming no change in precipitation), then the physiological effects of reducing stomatal opening, thus increasing WUE, would be dominant and recharge will remain largely stable, as the reduction in transpiration will compensate the increase in ground evaporation and evaporation from interception associated with higher VPD. However, this scenario is unlikely as the increase in CO_2_ and warming are largely linearly related (*42*) and a strong CO_2_ increase is accompanied by strong warming although with different regional magnitudes.

### Limits of interpretation

The presented results are based on a mechanistic ecohydrological model that conserves mass and energy and relies on well-established model components and parameterizations. However, some assumptions might dampen the variability in vegetation response. While the two simulated vegetation types, C_3_ grass and evergreen shrubs, can modify their leaf area and other carbon pools, the overall ground area occupied by vegetation (90%) is fixed, as are the vegetation rooting depth, soil properties and soil depth. A 2-m soil depth is a compromise between realism in describing the system and matching temporal dynamics of spring discharge (fig. S7). The vegetation parameters are also static, in lack of better information, which prevents the simulation of potential changes, due to local scale adaptation and plasticity of plant traits (*38*, *43*). CO_2_ feedbacks on belowground root dynamics are also limited to a change in fine-root biomass and thus soil-to-root conductance, while more complex root-associated ecological processes are not simulated (*44*, *45*). All these limitations imply that the study might underestimate the role of vegetation feedbacks. However, simulated GPP and LAI change by more than 50% across centuries already. In addition, the potential trends in wind speed (*46*) or changes in diurnal patterns and seasonality are not simulated by the weather generator, which might lead to further underestimation of the variability of ecohydrological responses. Anthropogenic activities, cultivars, and potential land-use changes are also not considered in this study. However, given the paramount role of precipitation changes in the past and of CO_2_-induced feedbacks in the future, which are driven by first-order hydrological and physiological dynamics, these limitations unlikely have substantial implications on the main conclusions.

## MATERIALS AND METHODS

### Study site and datasets for annual climate reconstruction

The analyzed region is representative of the rural area southwest of the city of Jerusalem (“Judea Group outcrops”) that recharges the regional Western Mountain Aquifer, which is the main groundwater source in the area (*16*). The region is in the transition between Mediterranean and semiarid climate; its rainy season lasts from October to April (annual mean precipitation in the period 1994–2019 is 470 mm), while months from May to September are dry and hot.

Hourly meteorological forcing in terms of precipitation, air temperature, relative humidity, shortwave radiation, wind speed, and atmospheric pressure were obtained from the meteorological station “Jerusalem Center” [31.78°N, 35.22°E, 810 m a.s.l. (above sea level)] of the Israel Meteorological Service (https://ims.gov.il/en/node/102) for the control period 1 September 1994 to 15 June 2019, which recorded a precipitation 8% larger than the proxy-based 4500-year average. These time series are used as forcing for the ecohydrological model during the “control period” and to parameterize the weather generator at the hourly scale.

Time series for mean annual precipitation and corresponding variance over the past 4500 years were recently reconstructed from an archive of sediment records that represent historical changes in the Dead Sea levels (*15*). This information was used to construct 2500 BCE to 1950 CE time series of annual precipitation that was then fed to the weather generator. Concurrently, time series of annual temperature was reconstructed for the period 2500 BCE to 849 CE and 850 CE to 1950 CE from the TraCE-21ka [Community Climate System Model version 3 (CCSM3), (*25*)] and Community Earth System Model Last Millennium Ensemble [CCSM4, (*26*)] models (www.earthsystemgrid.org/), respectively. Annual precipitation and daily temperature records derived from the observational ECA&D dataset [(*47*); www.ecad.eu/] were then used for the most recent years 1951–2000. Solar constant values to determine the incoming shortwave radiation at the top of the atmosphere were also varied across the 2500 BCE to 1885 CE period [(*24*); www2.mps.mpg.de/projects/sun-climate/]. The solar radiation at the surface was then computed by a weather generator (see below) assuming constant atmospheric properties except for CO_2_. The atmospheric CO_2_ data were taken from (*23*) for the period 2500 BCE to 1859 CE and are based on NASA reconstruction for the period 1860–1975 CE (https://data.giss.nasa.gov/) and on direct observations for the most recent years 1976–2019 CE (www.esrl.noaa.gov/gmd/dv/data/).

### Weather generator: Past climate and sensitivity analysis

To reconstruct hourly time series of meteorological data for the 2500 BCE to 2000 CE period and to create hypothetical meteorological time series for the sensitivity analysis, we used the stochastic weather generator AWE-GEN (*27*, *28*) that is designed to reproduce time series of climate variables based on local observations. It simulates precipitation, cloud cover, air temperature, vapor pressure, wind speed, atmospheric pressure, and shortwave incoming radiation. The weather generator can reproduce several statistics of meteorological variables across a wide range of temporal scales, including extremes and interannual variability. In the basic configuration, the weather generator can stochastically reproduce the present climate assuming stationarity. Here, AWE-GEN was first parameterized using the control period—25-year (1994–2019 CE) meteorological observations from the Jerusalem station. AWE-GEN can also account for additional information as annual values of precipitation, air temperature, and solar constant can be externally prescribed relaxing the assumption of stationarity. In this case, the seasonality and diurnal variability, as well as higher-order statistics, still depend on the original hourly scale parameterization, but the annual precipitation and mean air temperature will match the externally prescribed values. Because of cross-correlation among climatic variables, values of precipitation, air temperature, and solar constant might also affect other variables, e.g., cloud cover, solar radiation, and relative humidity, although seasonality and diurnal cycles are strongly linked to the original parameterization. The described method allows to stochastically simulate time series of the past 4500 years at the hourly scale combining 25 years of recent observations and the reconstructed annual values of precipitation, air temperature, and top of the atmosphere shortwave radiation.

In addition to the reconstruction of the historical climate, the parameters representing the control period were used to construct synthetic climate time series to force the ecohydrological model with modified Ta and CO_2_, but with prescribed long-term mean precipitation equal to 550 mm year^−1^ corresponding to the 1971–2000 CE period. As the increase in Ta and CO_2_ will be mostly linearly correlated in the future (*42*) but with local-scale variabilities, nine different scenarios were simulated where annual air temperature increases by up to 6°C in comparison to the 1971–2000’s CE mean annual temperature and CO_2_ level increases from 360 ppm (a reference value equal to the CO_2_ concentration in 1995) up to 920 ppm (fig. S8). To mediate the internal (stochastic) climate variability (*28*) in the other variables, nine realizations were simulated for every scenario for a total of 81 simulations.

### Ecohydrological modeling

The coupled water, energy, and carbon cycles were simulated with the mechanistic ecohydrological model Tethys-Chloris [T&C; (*21*, *22*)]. T&C resolves the mass and energy budgets at the land surface and describes physiological vegetation processes including photosynthesis, phenology, carbon allocation, and tissue turnover. A detailed model description is provided in previous studies, and the model has been extensively tested in several climates and ecosystems, including semiarid and Mediterranean ecosystems [e.g., (*21*, *22*, *32*, *48*–*50*)]. For this study, the vegetation parameterization (table S1) is taken from the Matta location [(*51*); 31.71°N, 35.07°E, 620 m a.s.l.], roughly 15 km southwest of Jerusalem. The local vegetation is composed of annual grasses covering roughly 50% of the area and dwarf shrubs, mostly *Sarcopoterium spinosum* that cover 40% of the area, with the remaining 10% assumed to be bare soil. Such a vegetation cover is common in the natural conditions in the region (*52*), although we acknowledge that part of the landscape is also occupied by olives, almonds, and pines, which may have deeper roots and a different ecohydrological response. For the case study of Matta, T&C provides realistic simulations of aboveground net primary productivity (ANPP) and reproduces the ANPP response to irrigation and precipitation exclusion experiments within the uncertainty of observations (*53*). Plant water stress is indicated by a β factor that accounts for the soil water potential integrated over the root zone and plant-specific water potential thresholds to control stomatal conductance, carbon allocation, and leaf turnover rates (*21*, *53*). Soil textural properties were obtained from the Soil-Grids map (*54*) using the average of the top 30 cm; specifically, we used 10% sand content and 23% clay content as characteristic values that were then converted into hydraulic properties using pedotransfer functions (*55*). The soil hydraulic properties were assumed to be vertically homogenous; in the model, the soil column was discretized using 20 vertical layers, with increasing depth from near the surface (1 cm) to the bedrock (25 cm), which was assumed to be at 2 m. A free drainage boundary condition was assumed at the interface between soil and bedrock. Fine-root biomass was distributed vertically using an exponential profile, and a maximum rooting depth of 35 cm was assumed for grass and shrubs (*53*). As the transfer of water through the bedrock to reach deep aquifers was not simulated, the 2-m bedrock depth is simply a compromise (obtained after a few trials) between considering recharge at a depth that is not influenced directly by surface processes (e.g., evaporation) but without imposing a too deep unrealistic soil profile. Water that percolates into the bedrock is generally assumed sheltered from ET and contributes to deep aquifer recharge.

### Model confirmation: Groundwater recharge estimates and remotely sensed LAI

Consistency of the response of the T&C model with stochastically generated meteorological forcing versus observations for the control period 1994–2019 CE was preliminary checked (fig. S9). The response in terms of the main ecohydrological variables was similar using both forcings, confirming the suitability of AWE-GEN in generating realistic climatic inputs for Jerusalem to be used in land surface models.

The LAI MODIS (Moderate Resolution Imaging Spectroradiometer) estimates (MOD15A2H v006; https://lpdaac.usgs.gov/products/mod15a2hv006/) for the period 2000–2019 CE of the Matta site were used to compare ecohydrological simulations of vegetation dynamics with independent observations. However, MODIS data should be interpreted cautiously as they are an indirect estimation of LAI only. The simulated LAI magnitude, in the range 0.7 to 1.4, and seasonality for the period of available remote sensing observations compare well with the MODIS LAI, although simulations show a more pronounced LAI peak at the end of the wet season in April in comparison to the remote sensing inference (fig. S7, A and B). Remotely sensed LAI also shows lower interannual variability in comparison to mechanistic simulations.

The 25 years (1994–2019 CE) of simulations are used to fit the relation between annual precipitation and annual groundwater recharge. We followed previous estimates based on discharge and precipitation data from five springs draining local perched aquifers in the region (*17*). These springs have different recharge areas, substrate geology, and mean precipitation. We note that these estimates are in the low envelope of the precipitation-recharge relations computed for the regional aquifer by other studies [e.g., (*16*, *18*)], but better represent the study area south of Jerusalem. The simulated annual precipitation–groundwater recharge (Pr-GR) relation is comparable with the estimates made in (*17*) and shows the expected exponential increase of annual groundwater recharge with annual precipitation (fig. S7C).

### Design of the ecohydrological numerical experiments

We used the hourly meteorological observations for the control period 1994–2019 CE, the AWE-GEN reconstructed hourly meteorological forcing from 2500 BCE to 2000 CE, and nine scenarios with modified Ta and CO_2_ to run the T&C model. Two additional scenarios were simulated, in which first, CO_2_ for the period 1790–2000 CE was fixed to the preindustrial level of 280 ppm to remove any CO_2_-induced effect; and second, leaf and other biomass pools were fixed to the preindustrial CO_2_ conditions imposing a predetermined LAI and biomass time series derived from the previous simulation but allowing CO_2_ to change, i.e., enabling for CO_2_ physiological effects but removing any feedback on aboveground and belowground vegetation growth.

In the other simulations, vegetation is dynamic in terms of carbon pools (e.g., LAI and fine-root biomass change throughout the simulation) but rooting depth and the ground fraction occupied by vegetation were fixed—by model construction. Model results were analyzed in terms of groundwater recharge, runoff, ET, LAI, and GPP. ET was further partitioned into its main components: transpiration, ground evaporation, and evaporation from interception. Hourly values were aggregated at the annual scale using hydrological (for validation) or calendar (for result analysis) year or at multiannual (e.g., 30 years) time scale as a simple average of the annual values.

## References

[1] J. S. Famiglietti, M. Rodell, Water in the balance. Science 340, 1300–1301 (2013). 23766323 10.1126/science.1236460

[2] Y. Wada, L. P. H. van Beek, C. M. van Kempen, J. Reckman, S. Vasak, M. F. P. Bierkens, Global depletion of groundwater resources. Geophys. Res. Lett. 37, L20402 (2010).

[3] J. S. Famiglietti, The global groundwater crisis. Nat. Clim. Chang. 4, 945–948 (2014).

[4] M. O. Cuthbert, T. Gleeson, N. Moosdorf, K. M. Befus, A. Schneider, J. Hartmann, B. Lehner, Global patterns and dynamics of climate-groundwater interactions. Nat. Clim. Chang. 9, 137–141 (2019).

[5] R. G. Taylor, B. Scanlon, P. Doll, M. Rodell, R. van Beek, Y. Wada, L. Longuevergne, M. Leblanc, J. S. Famiglietti, M. Edmunds, L. Konikow, T. R. Green, J. Y. Chen, M. Taniguchi, M. F. P. Bierkens, A. MacDonald, Y. Fan, R. M. Maxwell, Y. Yechieli, J. J. Gurdak, D. M. Allen, M. Shamsudduha, K. Hiscock, P. J. F. Yeh, I. Holman, H. Treidel, Ground water and climate change. Nat. Clim. Chang. 3, 322–329 (2013).

[6] J. H. Kim, R. B. Jackson, A global analysis of groundwater recharge for vegetation, climate, and soils. Vadose Zone J. 11, (2012).

[7] B. R. Scanlon, K. E. Keese, A. L. Flint, L. E. Flint, C. B. Gaye, W. M. Edmunds, I. Simmers, Global synthesis of groundwater recharge in semiarid and arid regions. Hydrol. Process. 20, 3335–3370 (2006).

[8] J. A. Davis, A. Kerezsy, S. Nicol, Springs: Conserving perennial water is critical in arid landscapes. Biol. Conserv. 211, 30–35 (2017).

[9] N. Peleg, E. Morin, H. Gvirtzman, Y. Enzel, Rainfall, spring discharge and past human occupancy in the Eastern Mediterranean. Clim. Chang. 112, 769–789 (2012).

[10] K. W. Butzer, G. H. Endfield, Critical perspectives on historical collapse. Proc. Natl. Acad. Sci. U.S.A. 109, 3628–3631 (2012). 22371580 10.1073/pnas.1114772109 PMC3309742

[11] H. Weiss, M. A. Courty, W. Wetterstrom, F. Guichard, L. Senior, R. Meadow, A. Curnow, The genesis and collapse of 3rd millennium north Mesopotamian civilization. Science 261, 995–1004 (1993). 17739617 10.1126/science.261.5124.995

[12] R. Ellenblum, *Collapse of the Eastern Mediterranean: Climate Change and the Decline of the East, 950-1072* (Cambridge Univ. Press, 2012).

[13] Y. Enzel, R. Bookman, D. Sharon, H. Gvirtzman, U. Dayan, B. Ziv, M. Stein, Late Holocene climates of the Near East deduced from Dead Sea level variations and modern regional winter rainfall. Quat. Res. 60, 263–273 (2003).

[14] C. Migowski, M. Stein, S. Prasad, J. F. W. Negendank, A. Agnon, Holocene climate variability and cultural evolution in the Near East from the Dead Sea sedimentary record. Quat. Res. 66, 421–431 (2006).

[15] E. Morin, T. Ryb, I. Gavrieli, Y. Enzel, Mean, variance, and trends of Levant precipitation over the past 4500 years from reconstructed Dead Sea levels and stochastic modeling. Quat. Res. 91, 751–767 (2019).

[16] N. Z. Dvory, Y. Livshitz, M. Kuznetsov, E. Adar, A. Yakirevich, The effect of hydrogeological conditions on variability and dynamic of groundwater recharge in a carbonate aquifer at local scale. J. Hydrol. 535, 480–494 (2016).

[17] N. Peleg, H. Gvirtzman, Groundwater flow modeling of two-levels perched karstic leaking aquifers as a tool for estimating recharge and hydraulic parameters. J. Hydrol. 388, 13–27 (2010).

[18] M. Weiss, H. Gvirtzman, Estimating ground water recharge using flow models of perched karstic aquifers. Ground Water 45, 761–773 (2007). 17973754 10.1111/j.1745-6584.2007.00360.x

[19] V. Marchionni, E. Daly, G. Manoli, N. J. Tapper, J. P. Walker, S. Fatichi, Groundwater buffers drought effects and climate variability in urban reserves. Water Resour. Res. 56, (2020).

[20] F. Orellana, P. Verma, S. P. Loheide, E. Daly, Monitoring and modeling water-vegetation interactions in groundwater-dependent ecosystems. Rev. Geophys. 50, RG3003 (2012).

[21] S. Fatichi, V. Y. Ivanov, E. Caporali, A mechanistic ecohydrological model to investigate complex interactions in cold and warm water-controlled environments: 1. Theoretical framework and plot-scale analysis. J. Adv. Model. Earth Syst. 4, M05002 (2012).

[22] S. Fatichi, C. Pappas, Constrained variability of modeledT: ETratio across biomes. Geophys. Res. Lett. 44, 6795–6803 (2017).

[23] E. Monnin, A. Indermuhle, A. Dallenbach, J. Fluckiger, B. Stauffer, T. F. Stocker, D. Raynaud, J. M. Barnola, Atmospheric CO2 concentrations over the last glacial termination. Science 291, 112–114 (2001). 11141559 10.1126/science.291.5501.112

[24] C. J. Wu, N. A. Krivova, S. K. Solanki, I. G. Usoskin, Solar total and spectral irradiance reconstruction over the last 9000 years. Astron. Astrophys. 620, A120 (2018).

[25] F. He, “Simulating transient climate evolution of the last deglaciation with CCSM3,” thesis, University of Wisconsin-Madison, Madison, WI (2011).

[26] B. L. Otto-Bliesner, E. C. Brady, J. Fasullo, A. Jahn, L. Landrum, S. Stevenson, N. Rosenbloom, A. Mai, G. Strand, Climate variability and change since 850 CE: An ensemble approach with the community earth system model. Bull. Am. Meteorol. Soc. 97, 735–754 (2016).

[27] S. Fatichi, V. Y. Ivanov, E. Caporali, Simulation of future climate scenarios with a weather generator. Adv. Water Resour. 34, 448–467 (2011).

[28] S. Fatichi, V. Y. Ivanov, A. Paschalis, N. Peleg, P. Molnar, S. Rimkus, J. Kim, P. Burlando, E. Caporali, Uncertainty partition challenges the predictability of vital details of climate change. Earths Future 4, 240–251 (2016).

[29] G. Shaffer, F. Lambert, In and out of glacial extremes by way of dust−climate feedbacks. Proc. Natl. Acad. Sci. 115, 2026–2031 (2018). 29440407 10.1073/pnas.1708174115 PMC5834668

[30] M. L. Roderick, F. Sun, W. H. Lim, G. D. Farquhar, A general framework for understanding the response of the water cycle to global warming over land and ocean. Hydrol. Earth Syst. Sci. 18, 1575–1589 (2014).

[31] R. J. Donohue, M. L. Roderick, T. R. McVicar, G. D. Farquhar, Impact of CO2 fertilization on maximum foliage cover across the globe’s warm, arid environments. Geophys. Res. Lett. 40, 3031–3035 (2013).

[32] S. Fatichi, S. Leuzinger, A. Paschalis, J. A. Langley, A. D. Barraclough, M. J. Hovenden, Partitioning direct and indirect effects reveals the response of water-limited ecosystems to elevated CO2. Proc. Natl. Acad. Sci. U.S.A. 113, 12757–12762 (2016). 27791074 10.1073/pnas.1605036113 PMC5111654

[33] R. J. Donohue, M. L. Roderick, T. R. McVicar, Y. Yang, A simple hypothesis of how leaf and canopy-level transpiration and assimilation respond to elevated CO2 reveals distinct response patterns between disturbed and undisturbed vegetation. J. Geophys. Res. Biogeosci. 122, 168–184 (2017).

[34] X. Lian, S. Piao, L. Z. X. Li, Y. Li, C. Huntingford, P. Ciais, A. Cescatti, I. A. Janssens, J. Peñuelas, W. Buermann, A. Chen, X. Li, R. B. Myneni, X. Wang, Y. Wang, Y. Yang, Z. Zeng, Y. Zhang, T. R. McVicar, Summer soil drying exacerbated by earlier spring greening of northern vegetation. Sci. Adv. 6, eaax0255 (2020). 31922002 10.1126/sciadv.aax0255 PMC6941915

[35] K. A. Novick, D. L. Ficklin, P. C. Stoy, C. A. Williams, G. Bohrer, A. C. Oishi, S. A. Papuga, P. D. Blanken, A. Noormets, B. N. Sulman, R. L. Scott, L. X. Wang, R. P. Phillips, The increasing importance of atmospheric demand for ecosystem water and carbon fluxes. Nat. Clim. Chang. 6, 1023–1027 (2016).

[36] Z. C. Zhu, S. L. Piao, R. B. Myneni, M. T. Huang, Z. Z. Zeng, J. G. Canadell, P. Ciais, S. Sitch, P. Friedlingstein, A. Arneth, C. X. Cao, L. Cheng, E. Kato, C. Koven, Y. Li, X. Lian, Y. W. Liu, R. G. Liu, J. F. Mao, Y. Z. Pan, S. S. Peng, J. Penuelas, B. Poulter, T. A. M. Pugh, B. D. Stocker, N. Viovy, X. H. Wang, Y. P. Wang, Z. Q. Xiao, H. Yang, S. Zaehle, N. Zeng, Greening of the Earth and its drivers. Nat. Clim. Chang. 6, 791–795 (2016).

[37] J. I. L. Morison, Sensitivity of stomata and water use efficiency to high CO2. Plant Cell Environ. 8, 467–474 (1985).

[38] T. Mastrotheodoros, C. Pappas, P. Molnar, P. Burlando, T. F. Keenan, P. Gentine, C. M. Gough, S. Fatichi, Linking plant functional trait plasticity and the large increase in forest water use efficiency. J. Geophys. Res. Biogeosci. 122, 2393–2408 (2017).

[39] A. Lavergne, H. Graven, M. G. De Kauwe, T. F. Keenan, B. E. Medlyn, I. C. Prentice, Observed and modelled historical trends in the water-use efficiency of plants and ecosystems. Glob. Chang. Biol. 25, 2242–2257 (2019). 30933410 10.1111/gcb.14634

[40] X. C. Zhou, E. Istanbulluoglu, E. R. Vivoni, Modeling the ecohydrological role of aspect-controlled radiation on tree-grass-shrub coexistence in a semiarid climate. Water Resour. Res. 49, 2872–2895 (2013).

[41] F. Giorgi, P. Lionello, Climate change projections for the Mediterranean region. Glob. Planet. Chang. 63, 90–104 (2008).

[42] S. I. Seneviratne, M. G. Donat, A. J. Pitman, R. Knutti, R. L. Wilby, Allowable CO2 emissions based on regional and impact-related climate targets. Nature 529, 477–483 (2016). 26789252 10.1038/nature16542

[43] M. V. Saha, T. M. Scanlon, P. D’Odorico, Examining the linkage between shrub encroachment and recent greening in water-limited southern Africa. Ecosphere 6, art156 (2015).

[44] C. M. Iversen, Digging deeper: Fine-root responses to rising atmospheric CO2 concentration in forested ecosystems. New Phytol. 186, 346–357 (2010). 20015070 10.1111/j.1469-8137.2009.03122.x

[45] M. Nie, M. Lu, J. Bell, S. Raut, E. Pendall, Altered root traits due to elevated CO2: A meta-analysis. Glob. Ecol. Biogeogr. 22, 1095–1105 (2013).

[46] T. R. McVicar, M. L. Roderick, R. J. Donohue, T. G. Van Niel, Less bluster ahead? Ecohydrological implications of global trends of terrestrial near-surface wind speeds. Ecohydrology 5, 381–388 (2012).

[47] A. Tank, J. B. Wijngaard, G. P. Konnen, R. Bohm, G. Demaree, A. Gocheva, M. Mileta, S. Pashiardis, L. Hejkrlik, C. Kern-Hansen, R. Heino, P. Bessemoulin, G. Muller-Westermeier, M. Tzanakou, S. Szalai, T. Palsdottir, D. Fitzgerald, S. Rubin, M. Capaldo, M. Maugeri, A. Leitass, A. Bukantis, R. Aberfeld, A. F. V. Van Engelen, E. Forland, M. Mietus, F. Coelho, C. Mares, V. Razuvaev, E. Nieplova, T. Cegnar, J. A. Lopez, B. Dahlstrom, A. Moberg, W. Kirchhofer, A. Ceylan, O. Pachaliuk, L. V. Alexander, P. Petrovic, Daily dataset of 20th-century surface air temperature and precipitation series for the European Climate Assessment. Int. J. Climatol. 22, 1441–1453 (2002).

[48] G. Manoli, V. Y. Ivanov, S. Fatichi, Dry-season greening and water stress in amazonia: The role of modeling leaf phenology. J. Geophys. Res.-Biogeosci. 123, 1909–1926 (2018).

[49] T. Mastrotheodoros, C. Pappas, P. Molnar, P. Burlando, G. Manoli, J. Parajka, R. Rigon, B. Szeles, M. Bottazzi, P. Hadjidoukas, S. Fatichi, More green and less blue water in the Alps during warmer summers. Nat. Clim. Chang. 10, 155–161 (2020).

[50] C. Pappas, S. Fatichi, P. Burlando, Modeling terrestrial carbon and water dynamics across climatic gradients: Does plant trait diversity matter? New Phytol. 209, 137–151 (2016). 26389742 10.1111/nph.13590

[51] K. Tielborger, M. C. Bilton, J. Metz, J. Kigel, C. Holzapfel, E. Lebrija-Trejos, I. Konsens, H. A. Parag, M. Sternberg, Middle-Eastern plant communities tolerate 9 years of drought in a multi-site climate manipulation experiment. Nat. Commun. 5, 5102 (2014). 25283495 10.1038/ncomms6102 PMC4205856

[52] O. Ackermann, A. M. Maeir, H. J. Bruins, Unique human-made catenary changes and their effect on soil and vegetation in the semi-arid Mediterranean zone: A case study on *Sarcopoterium spinosum* distribution near Tell eṡ-Ṡâfi/Gath, Israel. Catena 57, 309–330 (2004).

[53] A. Paschalis, S. Fatichi, J. Zscheischler, P. Ciais, M. Bahn, L. Boysen, J. F. Chang, M. De Kauwe, M. Estiarte, D. Goll, P. J. Hanson, A. B. Harper, E. Q. Hou, J. Kigel, A. K. Knapp, K. S. Larsen, W. Li, S. Lienert, Y. Q. Luo, P. Meir, J. Nabel, R. Ogaya, A. J. Parolari, C. H. Peng, J. Penuelas, J. Pongratz, S. Rambal, I. K. Schmidt, H. Shi, M. Sternberg, H. Q. Tian, E. Tschumi, A. Ukkola, S. Vicca, N. Viovy, Y. P. Wang, Z. N. Wang, K. Williams, D. H. Wu, Q. A. Zhu, Rainfall manipulation experiments as simulated by terrestrial biosphere models: Where do we stand? Glob. Chang. Biol. 26, 3336–3355 (2020). 32012402 10.1111/gcb.15024

[54] T. Hengl, J. M. de Jesus, G. B. M. Heuvelink, M. R. Gonzalez, M. Kilibarda, A. Blagotic, W. Shangguan, M. N. Wright, X. Y. Geng, B. Bauer-Marschallinger, M. A. Guevara, R. Vargas, R. A. MacMillan, N. H. Batjes, J. G. B. Leenaars, E. Ribeiro, I. Wheeler, S. Mantel, B. Kempen, SoilGrids250m: Global gridded soil information based on machine learning. PLOS ONE 12, e0169748 (2017). 28207752 10.1371/journal.pone.0169748 PMC5313206

[55] K. E. Saxton, W. J. Rawls, Soil water characteristic estimates by texture and organic matter for hydrologic solutions. Soil Sci. Soc. Am. J. 70, 1569–1578 (2006).

