# Supplementary material for: An ecohydrological journey of 4500 years reveals a stable but threatened precipitation–groundwater recharge relation around Jerusalem
Source: Sci Adv. 2021 Sep 10;7(37):eabe6303. doi: 10.1126/sciadv.abe6303 (PMC8442904; doi:10.1126/sciadv.abe6303)
Supplement: Supplementary file 1 — Figs. S1 to S9 Table S1 Legend for data S1 [file sciadv.abe6303_sm.pdf]

Science Advances
AAAS

Supplementary Materials for

An ecohydrological journey of 4500 years reveals a stable but threatened precipitation–groundwater recharge relation around Jerusalem

Simone Fatichi*, Nadav Peleg, Theodoros Mastrotheodoros,
Christoforos Pappas, Gabriele Manoli

*Corresponding author. Email: ceesimo@nus.edu.sg

Published 10 September 2021, *Sci. Adv.* **7**, eabe6303 (2021)
DOI: 10.1126/sciadv.abe6303

The PDF file includes:

Figs. S1 to S9
Table S1
Legend for data S1

Other Supplementary Material for this manuscript includes the following:

Data S1

Supplementary Materials

Supplementary Figures

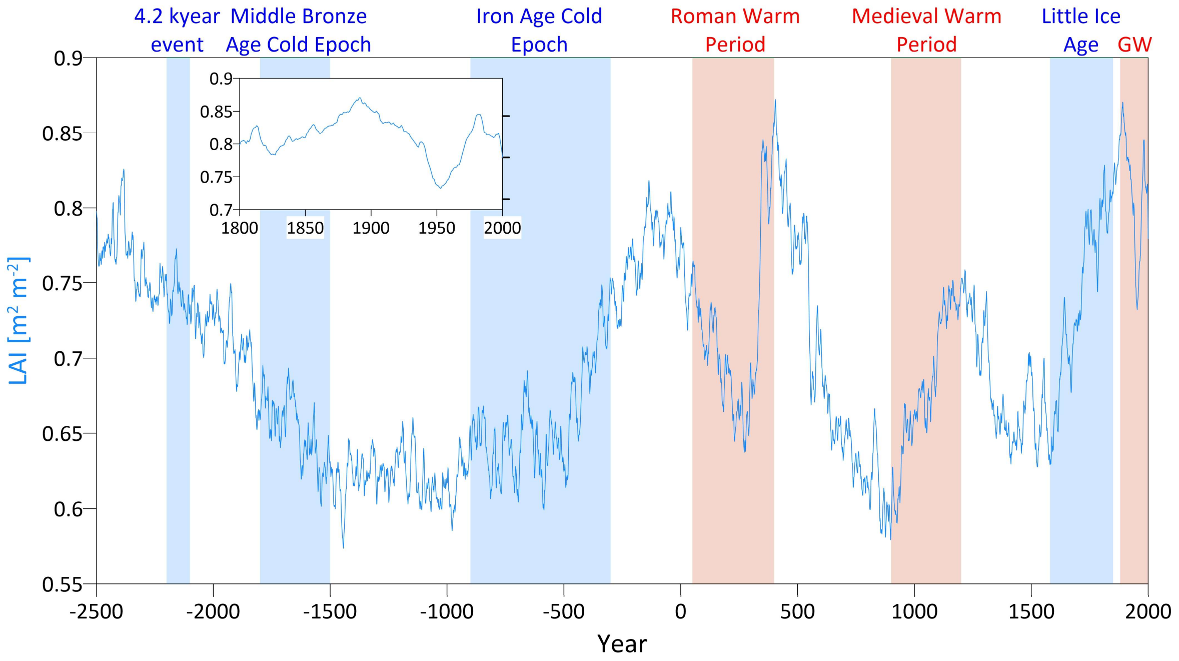

Figure S1. **Time series of simulated Leaf Area Index.** A 30-year moving average of simulated Leaf Area Index (LAI, blue line) between 2500 BC and 2000 AD. Major known cold and warm climatic epochs are framed in blue and red areas (respectively); GW – the current Global Warming. The last 200 years are zoomed in the inset.

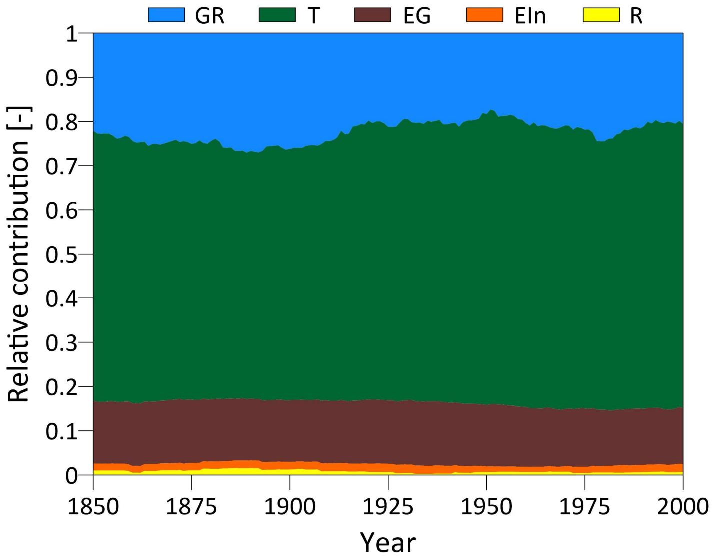

Figure S2. **Fractional hydrological budget components 1850 – 2000.** Partition of the hydrological budget components, normalized with precipitation, for the period 1850 – 2000 characterized by considerable anthropogenic greenhouse gas emissions. GR - groundwater recharge (127.7 mm y^{-1}), T- transpiration (344.6 mm y^{-1}), EG - ground evaporation (77.9 mm y^{-1}), EIn - evaporation from interception (9.2 mm y^{-1}), and R - runoff (4.8 mm y^{-1}). In parenthesis, the average value for the period.

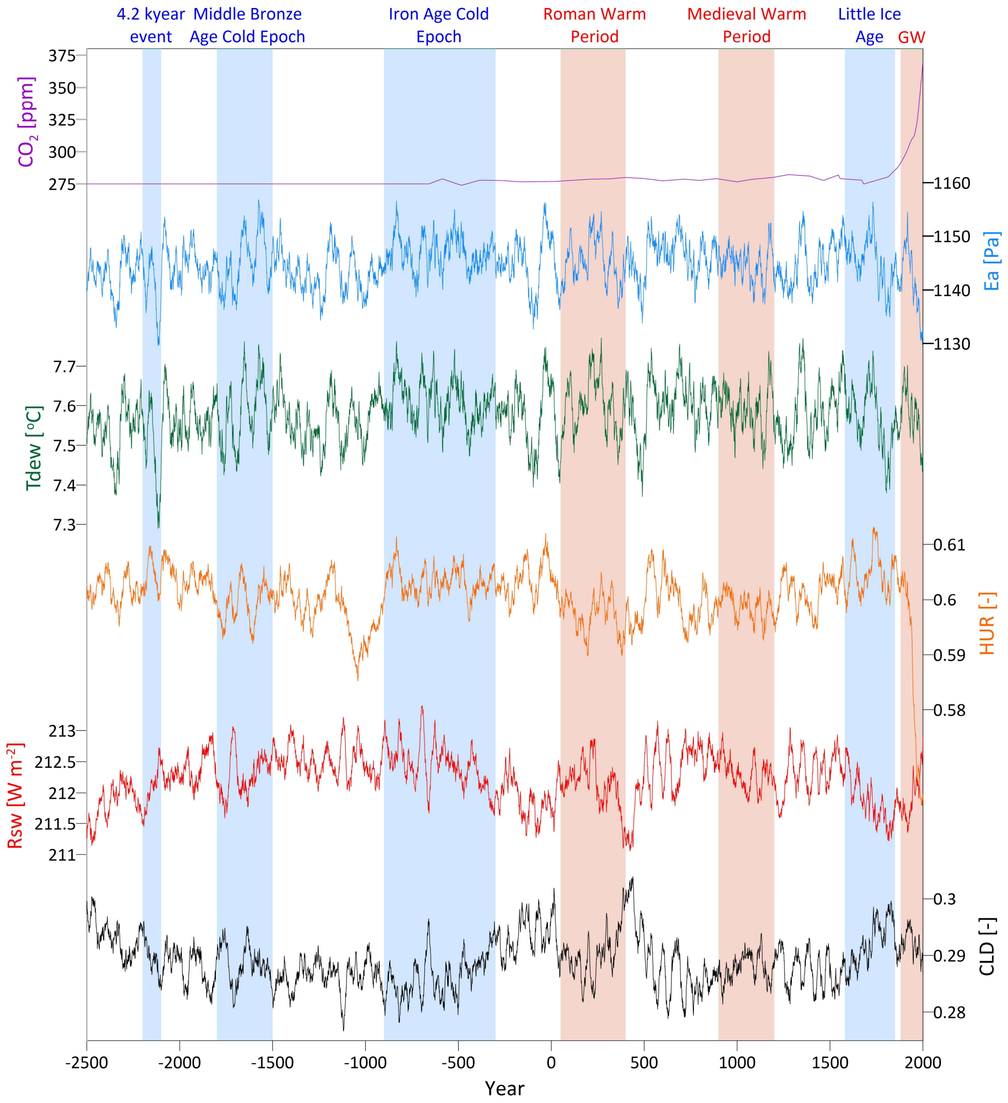

Figure S3. **Time series of meteorological forcing.** Reconstructed meteorological forcing (CO_2, purple line) and 30-year moving average of meteorological variables for the period between 2500 BC and 2000 AD simulated using AWE-GEN: vapor pressure, Ea, blue line; dew-point temperature, Tdew, green line; relative humidity, HUR, orange line; shortwave radiation, Rsw, red line; and cloud cover, CLD, black line. Major known cold and warm climatic epochs are framed in blue and red areas (respectively); GW is the current Global Warming.

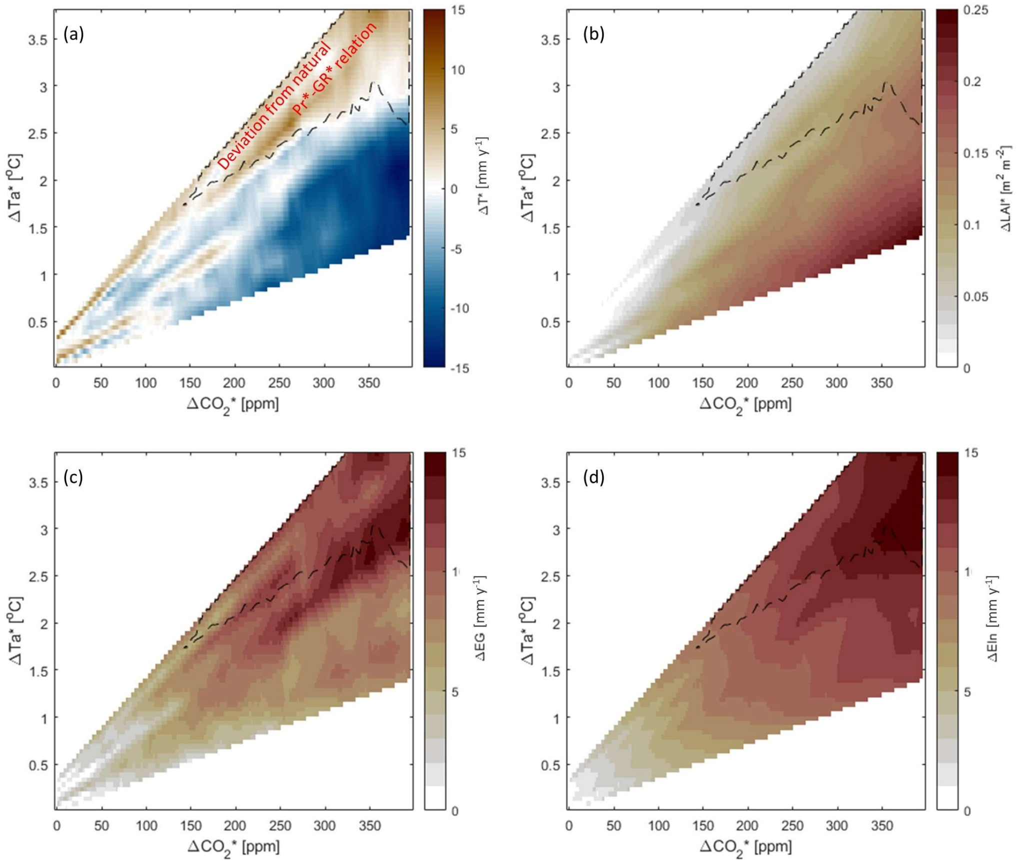

Figure S4. **Sensitivity to CO_2 and temperature increase of LAI and evapotranspiration fluxes**. Changes from present-day conditions (ΔTa*=0 and ΔCO_2*=0) for 30-year mean: (a) transpiration (T*); (b) leaf area index (LAI*); (c) ground evaporation (EG*); and (d) evaporation from interception (EIn*). Results are obtained for several combinations of increasing air temperature and CO_2 levels (Fig. S6). The dashed line marks in both subplots the lower boundary of the area where estimates of GR* exceed the 5-95th confidence interval of the natural Pr*-GR* relation.

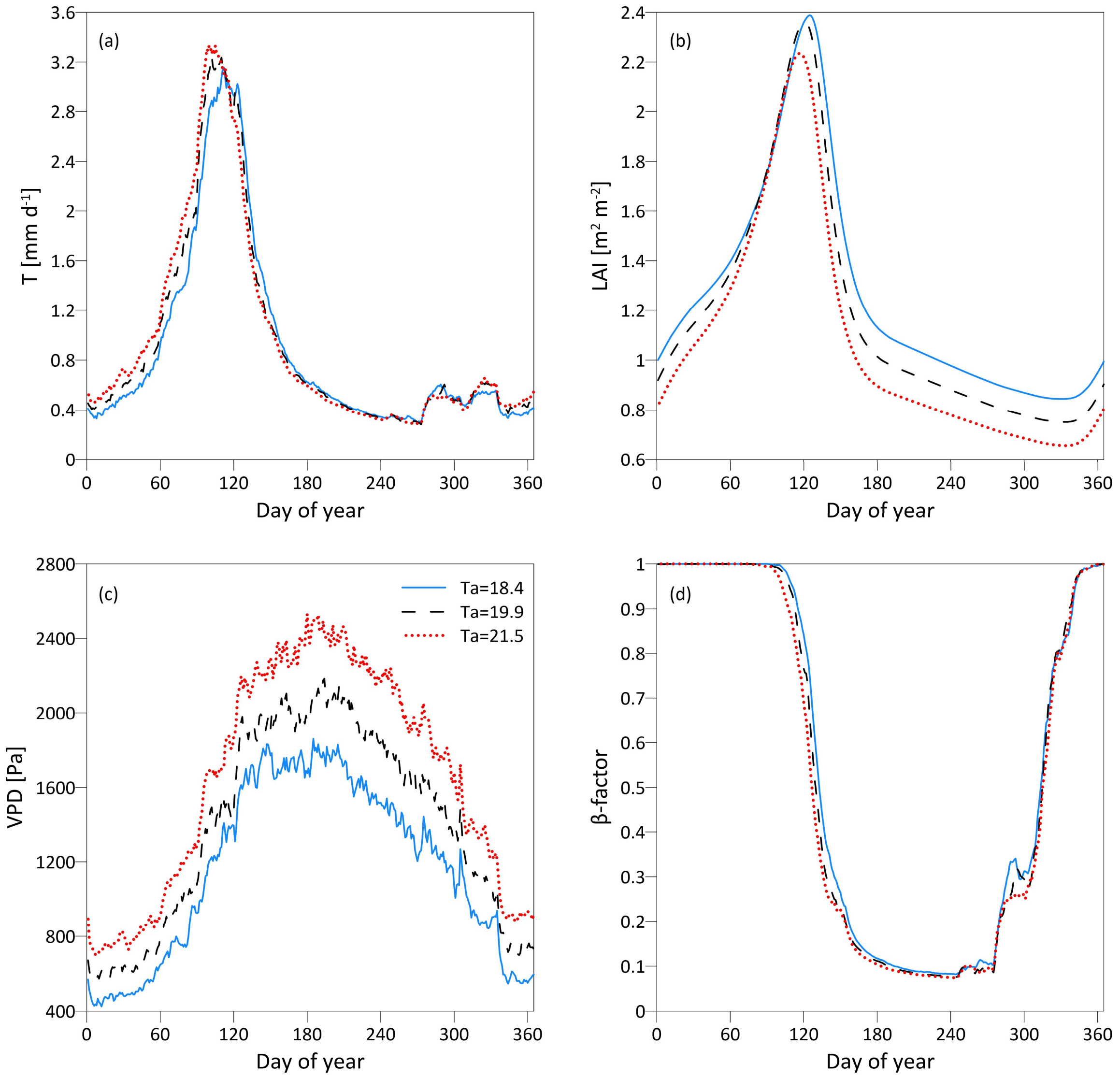

Figure S5. Seasonal cycle of ecohydrological variables. Average 30-year seasonal cycle of simulated (a) transpiration (T), (b) Leaf Area Index (LAI), (c) vapor pressure deficit (VPD), and (d) vegetation water stress factor β. β =1 means no stress and β=0 means maximum water stress. Results are extracted from the combinations of increasing air temperature and CO_2 levels (SC1, SC5, and SC9 - see Fig. S8) for a selected range of CO_2 levels (703 to 920 ppm).

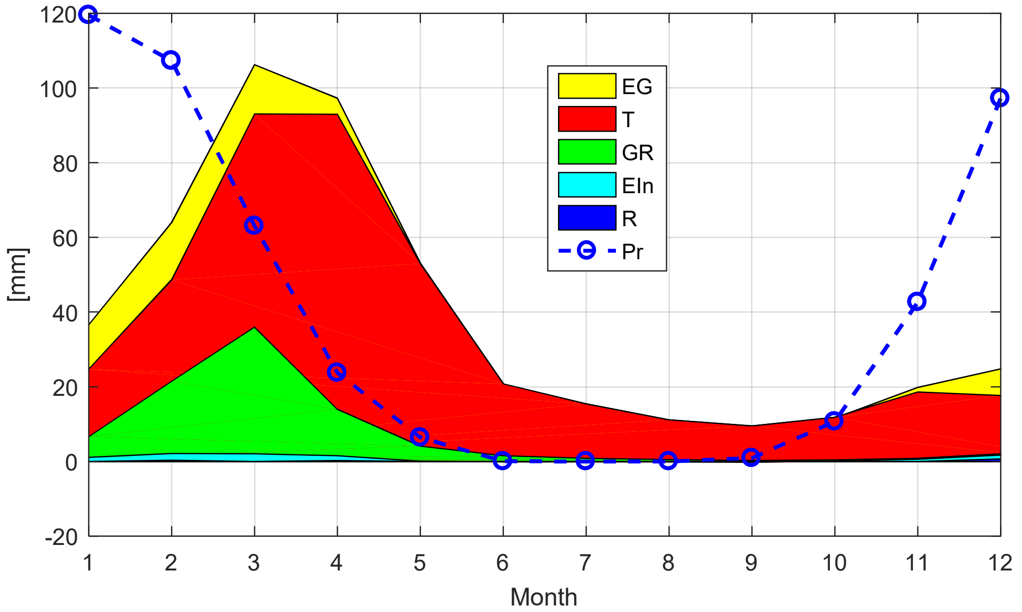

Figure S6. Seasonal water budget. Monthly values of precipitation (Pr), and of the sum of all the other hydrological budget components: transpiration (T), evaporation from interception (EIn), evaporation from ground (EG), runoff (R), and groundwater recharge (GR). Each component is indicated with a different color. Results are from the simulations forced with observed meteorological variables over the period 1994-2019.

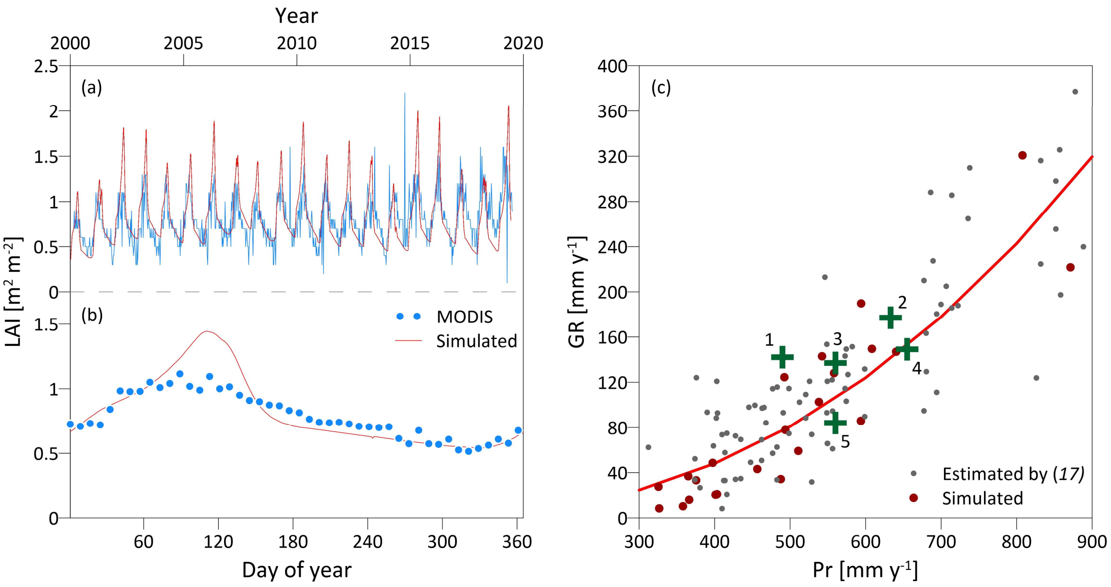

Figure S7. **Model validation**. (a) A comparison between observed (MODIS, blue) and simulated (red) leaf area index (LAI) for the period 2000-2019 and (b) seasonal average LAI. (c) Estimates of annual precipitation – groundwater recharge (Pr-GR) relation from (*17*) (grey dots), computed from precipitation - spring discharge relation for five springs located in the study area (see *9, 17* for details) for different periods. Green plus symbols represent the average annual Pr-GR relation for each spring: 1 – Delba (recharge area of 0.7 km^2), 2 – Haniya (2.73 km^2), 3 – Batir (1.06 km^2), 4 – Matwi (2.01 km^2), and 5 – Jamia (2.63 km^2). Red dots represent the simulated annual Pr-GR relation in the observational period 1994-2019, fitted with the non-linear equation $GR=4e\text{-}5Pr^{2.3}$; R^2=0.81 (red line).

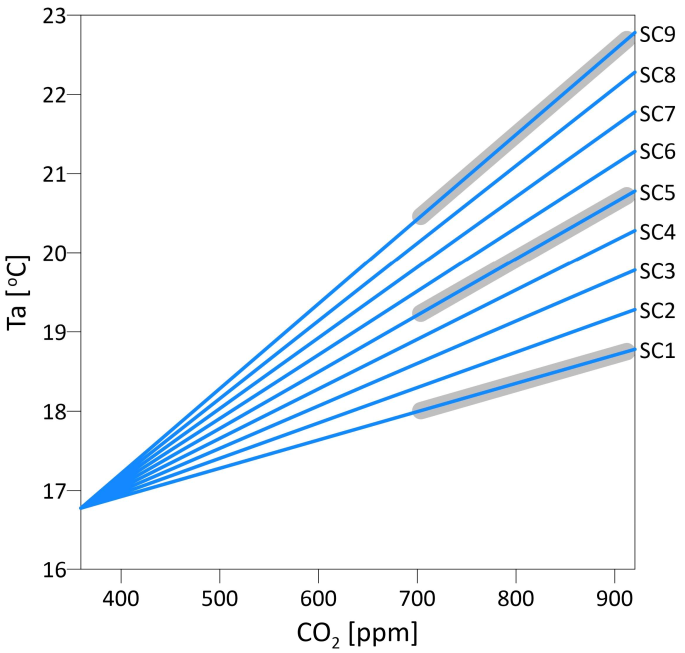

Figure S8. Sensitivity analysis scenarios. Nine CO_2 – air temperature (Ta) scenarios (labelled SC1 to SC9) are used as forcing for the climate sensitivity analysis (blue lines). Each temperature increase scenario consists of 945 simulated years (105 years linearly spanning the CO_2 levels between 360 and 920 ppm times 9 stochastic realizations). Grey areas in SC1, SC5, and SC9 mark the range used to extract the results presented in Fig. S5 (30 years x nine realizations per scenario).

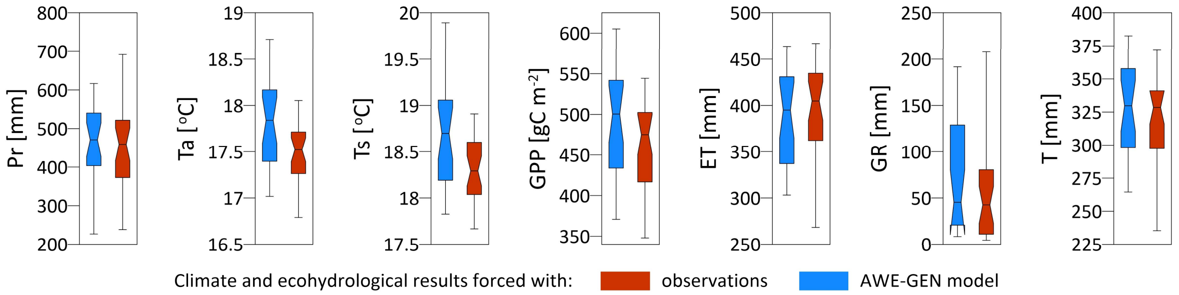

Figure S9. **Meteorological forcing and ecohydrological variables - observations versus AWE-GEN simulations**. The boxplots are showing the median (middle line), 25-75th percentile range (shaded area), and 5-95th percentile range (bounded with lines) of forcing climate (Pr – precipitation, Ta –air temperature) and simulated ecohydrological variables (Ts – surface temperature, GPP - gross primary production, ET – evapotranspiration, GR – groundwater recharge, T - transpiration). Variables are compared for simulations forced with the actual observations (1994-2019, blue) and the AWE-GEN simulation for the same period (26-year simulation).

Table S1. Model parameters of the T&C model used to simulate the C_3 grass (GRA) and evergreen shrubs (SHR) in this study.

**Parameter**	**Units**	**GRA**	**SHR**
Plant type	-	2	0
Root depth 95 percentile	mm	350	350
Specific interception of rainfall for unit leaf area	mm LAI^{-1}	0.2	0.2
Leaf characteristic dimension	cm	0.7	0.5
Canopy nitrogen decay coefficient	-	0.10	0.15
Intrinsic quantum efficiency	$umolCO_2$ $umolPhotons^{-1}$	0.081	0.081
Empirical coefficient for the role of vapor pressure in the biochemical model of photosynthesis	Pa	1200	1600
Empirical parameter connecting stomatal aperture and net assimilation	-	9	9
Minimum stomatal conductance	$molCO_2$ s^{-1} m^2	0.01	0.01
Photosynthesis pathway C_3 or C_4	[3-4]	3	3
Activation energy of photosynthesis (temperature dependence)	kJ mol^{-1}	0.649	0.649
Entropy factor of photosynthesis(temperature dependence)	kJ mol^{-1} K^{-1}	72	72
Mesophyll conductance	mol CO_2 s^{-1} m^{-2}	Inf	Inf
Scaling factor between J_{max} and $V_{c,max}$	umolEq $umolCO_2^{-1}$	2.1	2.1
Maximum Rubisco capacity at 25°C leaf level $V_{c,max}$	umol CO_2 m^2 s^{-1}	72	52
Water potential at the beginning of stomatal closure	MPa	-0.7	-0.7
Water potential at 50% of stomatal closure	MPa	-3.5	-5.5
Water potential at 50% impairment of growth and allocation control	Mpa	-1.5	-1.4
Water potential at 99% impairment of growth and allocation control	Mpa	-5.0	-7.0
Within canopy clumping factor	-	1	1
Specific leaf area	m^2 gC^{-1}	0.016	0.010
Leaf carbon nitrogen ratio	gC gN^{-1}	55	45
Maintenance respiration rate at 10°C	gC gN^{-1} day^{-1}	0.030	0.042
Growth respiration coefficient	-	0.25	0.25
Maximum leaf mortality factor for drought	day^{-1}	0.1	0.027
Factor of increasing mortality with cold	day °C	52	182
Leaf shed threshold for cold	°C	1	1
Fine root turnover rate	day	2000	1600
Sapwood to heartwood transfer rate	day	-	900
Critical leaf age	day	365	1200
Water stress threshold for leaf onset	-	0.99	0.99
Threshold temperature for leaf onset	°C	10.0	10.0
Days of maximum growth	d	25	15
Leaf area index for complete defoliation	m^2 LAI m^{-2} VEG^{-1}	0.05	0.001
Translocation rate from carbohydrate reserves	gC m^{-2} day^{-1}	1.3	0.4
Threshold for leaf onset: hours of light	h	9.0	9.0
Leaf to root biomass maximum ratio	-	0.8	1.0
Parameter for allocation to carbon reserves	[0-1]	1.0	1.0
Threshold for senescence: hours of light	h	9.0	9.0
Dead leaf fall turnover rate	day	50	50
Foliage cover decay factor for throughfall	-	0.75	0.75
Interception parameter	mm^{-1}	3.7	3.7
Interception drainage rate coefficient	mm	0.06	0.06

Data S1. **Annual values of simulated variables**. The supplementary data table contains weather generated meteorological variables and simulated ecohydrological variables for the period 2500 BC – 2000 AD. Annual values are obtained aggregating hourly values. Specifically, precipitation [mm], 2-m air temperature [°C], surface temperature [°C], atmospheric CO_2 concentration [ppm], Gross Primary Production (GPP) [gC m^{-2}], Leaf Area Index [m^2 m^{-2}], evapotranspiration [mm], transpiration [mm], ground evaporation [mm], evaporation from interception [mm], runoff [mm], and groundwater recharge [mm] are reported.
